# Evaluating the before operational stress program: comparing in-person and virtual delivery

**DOI:** 10.3389/fpsyg.2024.1382614

**Published:** 2024-07-25

**Authors:** Gabriela Ioachim, Nicole Bolt, Michelle Redekop, Andrew Wakefield, Andrii Shulhin, Jilani Dabhoya, Juliana M. B. Khoury, Kathy Bélanger, Sarah Williams, Tessa Chomistek, Taylor A. Teckchandani, Jill A. B. Price, Kirby Q. Maguire, R. Nicholas Carleton

**Affiliations:** ^1^Canadian Institute for Public Safety Research and Treatment-Institut Canadien de recherche et de traitement en sécurité publique (CIPSRT-ICRTSP), University of Regina/Université de Regina, Regina, SK, Canada; ^2^Anxiety and Illness Behaviours Lab, Department of Psychology, University of Regina, Regina, SK, Canada; ^3^Wayfound Mental Health Group Inc, Calgary, AB, Canada

**Keywords:** public safety personnel, stigma, resiliency, mental health training, emotional regulation, healthcare workers, virtual training, before operational stress

## Abstract

**Introduction:**

Public safety personnel (PSP) are at increased risk for posttraumatic stress injuries (PTSI). Before Operational Stress (BOS) is a mental health program for PSP with preliminary support mitigating PTSI. The current study compared the effectiveness of delivering BOS in-person by a registered clinician (i.e., Intensive) to virtually delivery by a trained clinician (i.e., Classroom).

**Methods:**

Canadian PSP completed the Intensive (*n* = 118; 61.9% male) or Classroom (*n* = 149; 50.3% male) program, with self-report surveys at pre-, post-, 1 month, and 4 months follow-ups.

**Results:**

Multilevel modelling evidenced comparable reductions in anxiety (*p* < 0.05, ES = 0.21) and emotional regulation difficulties (*p*s < 0.05, ESs = 0.20, 0.25) over time with no significant difference between modalities. Participants discussed benefits of the delivery modality they received.

**Discussion:**

The results support virtual delivery of the BOS program (Classroom) as an accessible mental health training option for PSP, producing effects comparable to in-person delivery by clinicians.

## 1 Introduction

Canadians rely on diverse public safety personnel (PSP; e.g., police, firefighters, paramedics) to ensure their safety and well-being (Mendicino and Blair, 2022). PSP are at increased risk for posttraumatic stress injuries (PTSI) (Carleton et al., 2020a; Heber et al., 2023) resulting from operational and organizational stressors (Carleton et al., 2020a). Almost half (44.5%) of PSP screen positive for one or more mental health disorders (Vig et al., 2020) and many report lifetime suicidal ideation (27.8%), planning (13.3%), and attempts (4.6%) (Carleton et al., 2018a). PTSI are associated with frequent exposures to potentially psychologically traumatic events (PPTEs; i.e., direct or indirect exposures to actual or threatened death, serious injury, or sexual violence) (Carleton et al., 2019a; Heber et al., 2023). PTSI are a substantial concern for PSP mental health and the Canadian government has recently developed an action plan to address challenges related to these mental health difficulties (Public Safety Canada, 2019).

There are several effective treatments for PTSI, such as cognitive behavioural therapy (CBT), that appear beneficial for PSP (Foa and Rothbaum, 2001; Ponniah and Hollon, 2009; Carleton, 2021; Hadjistavropoulos et al., 2021). Proactive solutions to mitigate PTSI also exist, often focusing on providing training or peer support to bolster resilience, minimize stigma, and develop individual stress management skills (Carleton et al., 2020b; Stelnicki et al., 2021). There has been relatively little research on such proactive efforts, with the available evidence suggesting small time-limited effects, necessitating recommendations for larger longitudinal research efforts (Anderson et al., 2020; Di Nota et al., 2022).

### 1.1 Overview of the before operational stress program

The Before Operational Stress (BOS) program was designed to provide access to effective evidence-based mental health training for PSP (McElheran et al., 2020; Stelnicki et al., 2021). The BOS programming is based on teaching core CBT skills and providing evidence informed learning content, taught by a clinician trained in CBT principles and familiar with PSP culture and treatment. BOS program combines theoretical and experiential learning procedures designed to improve resiliency, strengthen interpersonal relationships, and mitigate the effects of operational stressors. Participants complete one module per week for 8 weeks. The first six modules involve teaching participants to identify, understand, and navigate the connection between thoughts, emotions, physiological sensations, and behaviour. The final two modules focus on enhancing interpersonal relationships by teaching communication skills and empathy. Throughout the program, participants explore various mental health related topics to expand their knowledge of OSI and effective coping strategies. The program also emphasizes the importance of PSP maintaining healthy, authentic relationships, which can provide important mental health benefits for PSP as previous research suggests they are more likely to seek support from spouses and friends than professionals (Carleton et al., 2019b, 2020b; Nisbet et al., 2023).

The BOS Intensive program is facilitated in-person by a trained clinician in a group setting, following the one module per week for eight weeks model. Each 2 hour module-session is approximately evenly divided between a facilitator-led didactic component communicating program content, and 1 hour of group processing where participants share thoughts on the program content and discuss its application in their lives. A prior evaluation of the BOS Intensive program evidenced small but statistically significant improvements in PTSD symptoms and quality of life measures, increases in perceived social support, and reductions in mental health stigma associated with the training (Stelnicki et al., 2021). This evaluation was conducted with a smaller sample of participants who completed the training through the Original BOS Intensive (in-person) delivery modality and surveyed participants at four time points (before and after the training, and at 1 month and 3 month follow-ups). The available outcomes were associated with improved communication skills and more positive behaviours toward family members, as evidenced by qualitative responses.

The BOS Classroom program, consisting of 1 h sessions delivered virtually by a trained clinician, was developed to enhance training accessibility for individuals in areas lacking BOS-trained clinicians. The BOS Classroom modality removes the group processing aspect of the Intensive training to solely focus on delivery of the didactic component, therein shortening each module-session to approximately one hour. BOS Classroom became particularly important for maintaining training accessibility during public gathering restrictions imposed during the COVID-19 pandemic. The virtual training also helped address logistical access barriers by providing flexible timing and avoiding stigma barriers still prevalent in PSP workplaces (Rice et al., 2019; Hadjistavropoulos et al., 2021).

To date there has been no evaluation of the effectiveness of the BOS Classroom program and, by extension, no comparative assessment with the BOS Intensive program. Extant research indicates internet-based Cognitive Behavioural Therapy (ICBT) can be as effective as face-to-face CBT in treating a range of mental health challenges, offering similar therapeutic benefits, but with the added convenience of accessibility and flexibility (Andersson et al., 2019; Hadjistavropoulos et al., 2021; Thew et al., 2022). Previous evaluations of mindfulness training with military personnel demonstrated in-person training as producing better improvements in sleepiness, pain, and energy, than virtual training (Rice and Schroeder, 2021). The researchers suggested the in-person training benefits were supported by having longer sessions, stronger interpersonal bonds between the instructor and classmates (Rice and Schroeder, 2021), face-to-face interactions, greater accountability, and in-class participation (Rice et al., 2019).

### 1.2 The current study

The current study was designed to evaluate the effectiveness of the BOS Classroom program for improving mental health. The study hypotheses were: (1) participation in the BOS program would be associated with reductions in mental health symptoms, substance use, and mental health stigma, as well as increases in perceived social support, emotional regulation, resilience, and quality of life; and (2) the outcomes of the BOS Classroom program would be largely comparable to the BOS Intensive program.

We anticipate that the effect sizes in the BOS Classroom will be smaller than those in the BOS Intensive, although the direction of the effects is expected to be consistent across both modalities. This difference in effect sizes is expected due to the distinct characteristics of the Classroom modality, such as reduced group processing and shorter session durations. Although previous research suggests in-personal and virtual CBT training and treatment programs can have similar effectiveness (Spek et al., 2007; Andersson et al., 2016; Carlbring et al., 2018; Rice et al., 2018; Zhang et al., 2022; Alavi et al., 2023), BOS Classroom also features a reduced session time and omits the group processing component, which may affect the overall effectiveness of the training. The current study builds upon the findings of the previous independent evaluation of the BOS program (Stelnicki et al., 2021). Our study extends this work by comparing delivery modalities and employing a comprehensive mixed-methods approach, with a specific focus on contrasting the effectiveness of in-person and virtual delivery modalities.

## 2 Method

### 2.1 Procedure

The current study was approved by the University of Regina Institutional Research Ethics board (2018-191). Data were collected for two training modalities: BOS Intensive (i.e., in-person with group processing components) and BOS Classroom (i.e., virtual, didactic components only). The quasi-experimental design resulted from convenience samples arising from BOS Intensive groups being scheduled in-person until implementation of COVID-19 pandemic gathering restrictions, with BOS Classroom groups being scheduled after onset of the gathering restrictions. All participants completed a standardized intake interview prior to starting the BOS program, during which they were informed about potential voluntary participation in an independent research study evaluating the BOS training. Participants were screened prior to training for acute mental health distress or severe PTSD symptoms, and those in need were referred to therapy intervention resources. Participants who expressed interest were emailed a consent form along with a link to the first survey. Due to confidentiality assured during BOS training, data was only recorded from participants who expressed interest in the study, and the total number of participants in the BOS training at the time outside of study participation is not available. The research participation was voluntary, anonymous, and did not impact eligibility for the BOS program. Neither the clinical facilitators nor other group members were aware which members chose to participate in the study. No questions were mandatory, and participants could withdraw at any time without consequences for their training. Surveys were administered at four time points: pre-training, post-training, 1 month follow-up, and 4 months follow-up. All surveys contained the same measures, with pre-training also including sociodemographic measures, and post-training having an additional five open-ended questions requesting feedback on participant experiences in the training program. Although training recruitment was aimed at early career recruits, training was open to all career stages. As PSP will likely experience multiple exposure to PPTEs in their careers, BOS is intended as a proactive measure for any future exposures a PSP may experience.

### 2.2 Participants

A total of 267 participants were included in the study, of 118 which received the BOS Intensive (in-person) modality and 149 received the BOS Classroom (virtual) modality. Participants generally identified as male (61.9% Intensive, 50.3% Classroom), between 40 and 49 years old (39.0% Intensive, 32.9% Classroom), and married (71.2% Intensive, 72.5% Classroom). Program participants primarily resided in Western (56.8% Intensive; 46.3% Classroom) and Eastern Canada (33.9% Intensive; 43.6% Classroom). Most participants had graduated from a college program (35.6% Intensive, 19.5% Classroom) or completed a university degree (11.0% Intensive, 36.2% Classroom). Participants were typically firefighters (39.8% Intensive, 18.1% Classroom), paramedics (18.6% Intensive, 10.7% Classroom), or police (13.6% Intensive, 19.5% Classroom). Of the participants who answered pre- and post-training surveys, an approximate 15% returned for the four-month follow-up.

### 2.3 Measures

Study measures were chosen based on module content of the BOS program and to facilitate comparison with the BOS pilot study evaluation (Stelnicki et al., 2021).

#### 2.3.1 Alcohol use disorders identification test

The Alcohol Use Disorders Identification Test [AUDIT; Babor et al., 2001] is a 10-item self-report measure of potentially hazardous alcohol use. Participants are asked to rate each item on a 5-point Likert scale ranging from 0 (*never*) to 4 (*daily or almost daily*). Higher scores indicate more potentially hazardous alcohol use. The AUDIT is widely employed and has evidence of adequate psychometric properties (Reinert and Allen, 2002; de Meneses-Gaya et al., 2009; Peng et al., 2012). AUDIT scores can be used to screen for hazardous alcohol use (>7) and alcohol dependence (>15).

#### 2.3.2 Brief resiliency scale

The Brief Resiliency Scale (BRS; Smith et al., 2008) is a 6-item self-report measure of resilience. Participants rate each item on a 5-point Likert scale ranging from 1 (*strongly disagree*) to 5 (*strongly agree*). Items are summed to produce a total score, with higher scores indicating higher resiliency. The BRS has evidence of adequate internal consistency and test–retest reliability (Windle et al., 2011).

#### 2.3.3 Depression, anxiety, and stress scale

The Depression, Anxiety, and Stress Scale [DASS-21; Lovibond and Lovibond, 1995] is a 21-item self-report measure of designed to measure the negative emotional states of depression, anxiety, and stress. Each subscale consists of 7 items scored on a 5-point Likert scale ranging from 0 (*did not apply to me at all*) to 4 (*applied to me very much or most of the time*). Higher scores indicate greater symptom levels. The DASS-21 has evidence of adequate internal consistency, construct validity, and convergent and discriminant validity (Henry and Crawford, 2005). The DASS-21 subscale scores can be used to screen for clinically-significant depression (>20), anxiety (>14), and stress (>25).

#### 2.3.4 Difficulties in emotional regulation scale

The Difficulties in Emotional Regulation Scale (DERS; Gratz and Roemer, 2004) is a 36-item self-report measure of difficulties with emotional regulation. Participants rate each item on a 5-point Likert scale ranging from 1 (*almost never*) to 5 (*almost always*). All items can be summed to produce a total score, with higher scores indicating a greater degree of emotional dysregulation. Item subsets can be summed to produce subscales related to emotional responses (i.e., nonacceptance of emotional responses, lack of emotional awareness, limited access to emotional regulation strategies, and limited emotional clarity). The DERS has evidence of adequate reliability, as well as construct and concurrent validity (Gratz and Roemer, 2004; Bardeen et al., 2012; Fowler et al., 2014; Hallion et al., 2018), and may help predict responses to cognitive-behavioural therapy (Hallion et al., 2018).

#### 2.3.5 Opening minds survey for workplace attitudes

The Opening Minds Survey for Workplace Attitudes (OMSWA; Szeto et al., 2013) is an 11-item self-report measure of attitudes towards people with mental illness. Participants rate each item on a 5-point Likert scale ranging from 1 (*strongly disagree*) to 5 (*strongly agree*). Items are summed to produce a total score with higher scores indicating a higher degree of stigma in the workplace. The OMSWA has been widely employed by the Mental Health Commission of Canada to measure stigma (Krakauer et al., 2020) and with evidence of adequate factor validity for the 9-item version (Boehme et al., 2022).

#### 2.3.6 PTSD checklist for DSM-5

The PTSD Checklist for DSM-5 (PCL-5; Weathers et al., 2013) PCL-5 is a 20-item self-report measure used to assess PTSD symptoms in the past month. Participants who identify exposure to at least one PPTE are then asked to select which exposure has caused them the most difficulty recently and answer questions regarding how much they have been bothered by different aspects of that event over the past month. Participants rate each item on a 5-point Likert scale ranging from 0 (*not at all*) to 4 (*extremely*). All item scores can be summed to produce a total score, and subsets of items can be summed to produce subscale scores reflecting DSM-5 symptom clusters B (re-experiencing/re-living), C (avoiding reminders of the incident), D (negative thoughts and mood), and E (hyper arousal/alertness). Higher scores indicate greater symptom levels. The PCL-5 has evidence of adequate reliability, as well as structural, convergent, and discriminant validity (Blevins et al., 2015; Ashbaugh et al., 2016). A positive screen for probable PTSD can be made for participants who endorse symptoms in each PTSD cluster and exceed the minimum clinical cutoff of 32 for the total PCL-5 score.

#### 2.3.7 Social provisions scale

The Social Provisions Scale (SPS10; Cutrona and Russell, 1987) is a 10-item self-report measure of extent of social support. Participants rate each item on a 4-point Likert-type scale ranging from 1 (*strongly disagree*) to 4 (*strongly agree*). All items can be summed to produce a total score, and the SPS10 has evidence of adequate reliability and convergent validity (Gottlieb and Bergen, 2010).

#### 2.3.8 WHO quality of life-BREF (WHOQOL-BREF)

The WHO Quality of Life-BREF (WHOQOL-BREF; WHOQOL Group, 1998) is a 25-item self-report measure of quality of life. Participants rate each item on a 5-point Likert scale ranging from 1 (*not at all*) to 5 (*an extreme amount*). The mean of all items is calculated to produce a total score, with item subset means calculated to produce subscale scores describing each of four domains: physical health, psychological health, social relationships, and environmental quality of life. We analyzed domains 2, 3, and 4, which relate to psychological and social quality of life. Higher mean scores indicate greater quality of life. The scale has evidence of adequate psychometric properties (Skevington et al., 2004).

### 2.4 Qualitative data and analyses

Participants were asked five open–ended questions at the end of the post-training survey (i.e., upon BOS program completion): (1) What has been the most helpful aspect of BOS for you?; (2) What has been the least helpful aspect of BOS for you?; (3) Has anything gotten better for you as a result of BOS? If so, please describe; (4) Has anything gotten worse for you as a result of BOS? If so, please describe; and (5) Please use the space below to provide any other comments you would like about your participation in BOS. Of the initial participant responses, 9 (*n* = 9) were excluded due to missing data, resulting in 30 Intensive and 45 Classroom responders, totaling 75 (*n* = 75) participants. Responses were anonymized and analyzed in NVivo 12. Two authors (MR and AW) used the coding procedures outlined in Miles et al. (2018) to separately inductively analyze the data. The same authors then collaborated to create broad themes by comparing their two code lists, producing a master coding framework, and recoding the data. A comparison query was run to determine the degree of overlap between coders (calculated at 80% agreement with a Cohen’s Kappa of 0.40, suggesting a fair level of agreement beyond what might be expected from chance) and to reach consensus on codes of disagreement (i.e., what theme best captured codes of discrepancy between authors). The authors then re-visited the coded data and addressed discrepancies in themes to achieve 100% agreement and co-constructed definitions to closely represent participant experiences by discussing the coded themes and the associated interrelationships. A matrix coding query was used to analyze the differences in theme references between Intensive and Classroom modalities and to clarify final participant counts for each theme.

### 2.5 Quantitative analyses

Descriptive analyses provided information about the sociodemographic characteristics of participants in the Intensive and Classroom modalities (Table 1). Descriptive analyses characterized mental health and resilience scale variables over time (Table 2). Generalized Estimating Equations were used to assess whether the number of participants screening positive for a particular mental health challenge changed statistically significantly over time (Table 3) (Andreski et al., 1998; Heine et al., 2011). Modality was included as a factor to assess whether the number of participants with a positive screen differed between Intensive and Classroom modalities. Interaction effects were initially tested, but none were statistically significant (all *p*s > 0.05), so none were retained for the final model.

**Table 1:** Public safety personnel demographics by delivery modality.

	Intensive	Classroom
	Total % (*n*)	Total % (*n*)
Sex		
Male	61.9 (73)	50.3 (75)
Female	28.0 (33)	46.3 (69)
Other	^	^
Age		
19–29	^	4.0 (6)
30–39	26.3 (31)	22.8 (34)
40–49	39.0 (46)	32.9 (49)
50–59	21.2 (25)	29.5 (44)
Marital Status		
Single	10.2 (12)	12.1 (18)
Married/Common-Law	71.2 (84)	72.5 (108)
Divorced/Separated/Widowed	5.9 (7)	9.4 (14)
Rather Not Say	^	^
Province of Residence		
Western Canada (BC, AB, SK, MB)	56.8 (67)	46.3 (69)
Eastern Canada (ON, QC)	33.9 (40)	43.6 (65)
Atlantic Canada (PEI, NS, NB, NFL)	^	^
Northern Territories (YK, NWT, NVT)	^	^
Education		
High School or High School Equivalent	10.2 (12)	7.4 (11)
Partial College Education	11.0 (13)	8.1 (12)
Graduated 2/3-Year College Program	35.6 (42)	29.5 (44)
University Degree/4-Year College	11.0 (26)	36.2 (54)
Completed Graduate School	7.6 (9)	12.8 (19)
Other	^	^
Public Safety Personnel Category		
Firefighter	39.8 (47)	18.1 (27)
Paramedic	18.6 (22)	10.7 (16)
Municipal/Provincial Police	13.6 (16)	19.5 (29)
Royal Canadian Mounted Police	11.0 (13)	6.0 (9)
Crown Prosecutor	^	^
Foreign Service Officer	^	^
Communication Officials	^	^
Other—specified (dispatcher, prosecutor, intelligence, emergency response, investigator, union of solicitor general)	10.2 (12)	16.8 (25)
Other—not listed	^	28.2 (42)

Cells marked with “^” contain five or fewer participant responses.

**Table 2:** Self-report mental health measure metrics by time.

Variable	Delivery modality	Time															
		Pre-training (*n* = 216)				Post-training (*n* = 87)				1 Month post-training (*n* = 60)				4 Months post-training (*n* = 30)			
		(*n*)		M	(SD)	(*n*)		M	(SD)	(*n*)		M	(SD)	(*n*)		M	(SD)
Probable PTSD-PCL5 Total Score	Total	98.6	(213)	20.15	(16.73)	97.7	(85)	21.42	(19.92)	95.0	(57)	20.65	(14.31)	96.7	(29)	21.90	(17.40)
	Intensive	41.2	(89)	22.09	(17.33)	49.4	(43)	23.30	(19.72)	35.0	(21)	18.86	(11.37)	43.3	(13)	19.23	(14.60)
	Classroom	57.4	(124)	18.76	(16.21)	48.3	(42)	19.50	(20.18)	60.0	(36)	21.69	(15.84)	53.3	(16)	24.06	(19.58)
Probable PTSD-PCL5 Cluster B	Total	100.0	(216)	4.77	(4.36)	100.0	(87)	4.79	(4.88)	96.7	(58)	4.62	(3.91)	96.7	(29)	5.31	(4.41)
	Intensive	42.1	(91)	5.16	(4.89)	50.6	(44)	5.09	(5.00)	35.0	(21)	4.05	(3.04)	43.3	(13)	5.08	(3.82)
	Classroom	57.9	(125)	4.48	(3.94)	49.4	(43)	4.49	(4.78)	61.7	(37)	4.95	(4.33)	53.3	(16)	5.50	(4.95)
Probable PTSD-PCL5 Cluster C	Total	100.0	(216)	2.28	(2.22)	98.9	(86)	2.21	(2.36)	96.7	(58)	2.45	(2.26)	100.0	(30)	2.37	(2.27)
	Intensive	42.1	(91)	2.36	(2.17)	49.4	(43)	2.42	(2.14)	35.0	(21)	2.00	(1.92)	43.3	(13)	1.92	(2.06)
	Classroom	57.9	(125)	2.22	(2.26)	49.4	(43)	2.00	(2.57)	61.7	(37)	2.70	(2.43)	56.7	(17)	2.71	(2.42)
Probable PTSD-PCL5 Cluster D	Total	99.1	(214)	6.84	(6.44)	100.0	(87)	7.44	(7.65)	95.0	(57)	6.81	(5.73)	100.0	(30)	7.03	(6.24)
	Intensive	41.7	(90)	7.42	(6.27)	50.6	(44)	8.30	(7.83)	35.0	(21)	6.57	(4.96)	43.3	(13)	5.54	(5.32)
	Classroom	57.4	(124)	6.41	(6.55)	49.4	(43)	6.56	(7.45)	60.0	(36)	6.94	(6.21)	56.7	(17)	8.18	(6.80)
Probable PTSD-PCL5 Cluster E	Total	99.5	(215)	6.19	(5.45)	98.9	(86)	7.02	(6.28)	96.7	(58)	6.55	(4.90)	100.0	(30)	7.77	(6.10)
	Intensive	41.7	(90)	7.08	(5.66)	50.6	(44)	7.77	(6.22)	35.0	(21)	6.24	(3.92)	43.3	(13)	6.69	(4.87)
	Classroom	57.9	(125)	5.55	(5.22)	48.3	(42)	6.24	(6.32)	61.7	(37)	6.73	(5.41)	56.7	(17)	8.59	(6.92)
Depression-DASS21-Dep	Total	94.9	(205)	8.21	(8.72)	95.4	(83)	9.98	(10.16)	100.0	(60)	9.13	(8.51)	93.3	(28)	8.29	(8.41)
	Intensive	38.4	(83)	8.75	(8.14)	46.0	(40)	10.30	(9.01)	38.3	(23)	8.35	(8.50)	40.0	(12)	5.50	(6.61)
	Classroom	56.5	(122)	7.85	(9.10)	49.4	(43)	9.67	(11.22)	61.7	(37)	9.62	(8.60)	53.3	(16)	10.38	(9.19)
Anxiety-DASS21-Anx	Total	95.4	(206)	5.82	(7.22)	96.6	(84)	6.62	(8.34)	100.0	(60)	4.97	(5.90)	93.3	(28)	6.64	(7.30)
	Intensive	38.4	(83)	6.29	(8.14)	48.3	(42)	6.52	(7.60)	38.3	(23)	4.52	(5.05)	40.0	(12)	6.33	(6.87)
	Classroom	56.9	(123)	5.50	(6.54)	48.3	(42)	6.71	(9.12)	61.7	(37)	5.24	(6.42)	53.3	(16)	6.88	(7.83)
Stress-DASS21Str	Total	94.9	(205)	12.01	(9.01)	96.6	(84)	12.60	(10.05)	100.0	(60)	12.97	(9.25)	93.3	(28)	11.86	(9.51)
	Intensive	38.0	(82)	12.85	(9.04)	48.3	(42)	12.90	(8.81)	38.3	(23)	11.65	(7.69)	40.0	(12)	10.83	(7.79)
	Classroom	56.9	(123)	11.45	(8.98)	48.3	(42)	12.29	(11.26)	61.7	(37)	13.78	(10.12)	53.3	(16)	12.63	(10.80)
Emotional regulation-DERS36 Total Score	Total	88.9	(192)	78.76	(22.80)	93.1	(81)	79.57	(25.51)	96.7	(58)	76.14	(23.83)	93.3	(28)	76.18	(25.02)
	Intensive	37.5	(81)	81.37	(21.96)	47.1	(41)	84.56	(23.61)	36.7	(22)	77.91	(20.97)	40.0	(12)	71.08	(20.85)
	Classroom	51.4	(111)	76.86	(23.30)	46.0	(40)	74.45	(26.65)	60.0	(36)	75.06	(25.64)	53.3	(16)	80.00	(27.78)
Emotional regulation-DERS36 Non-Acceptance	Total	93.1	(201)	13.10	(5.81)	96.6	(84)	12.67	(6.18)	98.3	(59)	12.68	(5.95)	93.3	(28)	12.50	(6.35)
	Intensive	37.5	(81)	13.75	(6.00)	48.3	(42)	13.21	(6.19)	36.7	(22)	14.36	(6.39)	40.0	(12)	12.92	(6.86)
	Classroom	55.6	(120)	12.66	(5.66)	48.3	(42)	12.12	(6.19)	61.7	(37)	11.68	(5.52)	53.3	(16)	12.19	(6.15)
Emotional regulation-DERS36 Awareness	Total	93.1	(201)	16.97	(5.69)	95.4	(83)	16.71	(5.67)	96.7	(58)	16.05	(4.74)	93.3	(28)	15.00	(4.92)
	Intensive	38.0	(82)	17.21	(5.73)	48.3	(42)	17.71	(5.22)	36.7	(22)	16.18	(4.17)	40.0	(12)	14.25	(4.97)
	Classroom	55.1	(119)	16.81	(5.68)	47.1	(41)	15.68	(5.98)	60.0	(36)	15.97	(5.11)	53.3	(16)	15.56	(4.97)
Emotional regulation-DERS36 Strategy	Total	93.1	(201)	14.74	(5.98)	93.1	(81)	14.91	(6.62)	98.3	(59)	14.36	(6.08)	93.3	(28)	14.86	(6.68)
	Intensive	37.5	(81)	15.17	(5.44)	47.1	(41)	15.83	(6.23)	36.7	(22)	14.55	(5.51)	40.0	(12)	13.17	(4.13)
	Classroom	55.6	(120)	14.44	(6.32)	46.0	(40)	13.98	(6.96)	61.7	(37)	14.24	(6.47)	53.3	(16)	16.13	(7.98)
Emotional regulation-DERS36 Clarity	Total	94.9	(205)	10.81	(3.75)	96.6	(84)	11.50	(4.16)	98.3	(59)	10.32	(3.19)	93.3	(28)	10.43	(3.68)
	Intensive	38.0	(82)	11.07	(3.79)	48.3	(42)	12.62	(4.24)	36.7	(22)	10.27	(2.51)	40.0	(12)	9.83	(4.09)
	Classroom	56.9	(123)	10.63	(3.73)	48.3	(42)	10.38	(3.80)	61.7	(37)	10.35	(3.56)	53.3	(16)	10.88	(3.40)
Quality of life—WHOQOL Domain 2	Total	93.1	(201)	64.11	(17.94)	93.1	(81)	63.07	(17.96)	93.3	(56)	63.02	(19.79)	93.3	(28)	62.75	(20.34)
	Intensive	37.0	(80)	63.29	(17.15)	48.3	(42)	60.00	(15.97)	33.3	(20)	65.85	(15.99)	40.0	(12)	66.75	(17.64)
	Classroom	56.0	(121)	64.65	(18.50)	44.8	(39)	66.38	(19.55)	60.0	(36)	61.44	(21.66)	53.3	(16)	59.75	(22.23)
Quality of life—WHOQOL Domain 3	Total	93.1	(201)	62.74	(19.69)	93.1	(81)	61.52	(20.60)	93.3	(56)	61.16	(21.71)	93.3	(28)	64.93	(25.15)
	Intensive	37.0	(80)	60.36	(20.02)	48.3	(42)	60.71	(17.52)	33.3	(20)	62.90	(17.96)	40.0	(12)	70.25	(21.76)
	Classroom	56.0	(121)	64.31	(19.40)	44.8	(39)	62.38	(23.68)	60.0	(36)	60.19	(23.73)	53.3	(16)	60.94	(27.42)
Quality of life—WHOQOL Domain 4	Total	93.1	(201)	74.80	(13.76)	93.1	(81)	73.38	(15.02)	93.3	(56)	72.02	(15.74)	93.3	(28)	76.79	(16.89)
	Intensive	37.0	(80)	73.44	(13.39)	48.3	(42)	71.57	(13.17)	33.3	(20)	73.90	(13.75)	40.0	(12)	77.25	(16.34)
	Classroom	56.0	(121)	75.70	(13.98)	44.8	(39)	75.33	(16.74)	60.0	(36)	70.97	(16.84)	53.3	(16)	76.44	(17.82)
Resilience—BRS total score	Total	91.7	(198)	3.65	(0.70)	93.1	(81)	3.63	(0.76)	91.7	(55)	3.69	(0.76)	93.3	(28)	3.57	(0.87)
	Intensive	36.1	(78)	3.54	(0.72)	48.3	(42)	3.50	(0.70)	35.0	(21)	3.72	(0.75)	40.0	(12)	3.75	(0.78)
	Classroom	55.6	(120)	3.72	(0.68)	44.8	(39)	3.77	(0.80)	56.7	(34)	3.67	(0.78)	53.3	(16)	3.44	(0.93)
Support—SPS10 total score	Total	88.0	(190)	32.51	(5.63)	86.2	(75)	32.09	(6.69)	85.0	(51)	33.80	(4.75)	93.3	(28)	32.54	(6.39)
	Intensive	34.7	(75)	32.21	(5.65)	44.8	(39)	31.90	(5.85)	30.0	(18)	33.67	(4.55)	40.0	(12)	34.17	(4.30)
	Classroom	53.2	(115)	32.70	(5.64)	41.4	(36)	32.31	(7.59)	55.0	(33)	33.88	(4.92)	53.3	(16)	31.31	(7.50)
Stigma—OMSWA total score	Total	76.9	(166)	14.90	(4.70)	70.1	(61)	15.21	(5.16)	70.0	(42)	15.62	(6.47)	76.7	(23)	14.83	(5.71)
	Intensive	32.9	(71)	16.06	(4.69)	35.6	(31)	16.90	(5.39)	26.7	(16)	14.31	(4.00)	33.3	(10)	14.80	(5.27)
	Classroom	44.0	(95)	14.04	(4.54)	34.5	(30)	13.47	(4.34)	43.3	(26)	16.42	(7.56)	43.3	(13)	14.85	(6.24)

Total percentages may not sum to 100 due to non-response or responding “other.” PCL-5, posttraumatic stress disorder checklist for DSM-5 (Subscale cluster B—re-experiencing, cluster C—avoiding, cluster D, negative thoughts, cluster E—arousal/alertness); DASS, depression, anxiety, and stress scale; DERS, difficulties in emotion regulation scale (subscales for non-acceptance of emotional responses, lack of emotional awareness, limited access to emotional regulation strategies, lack of emotional clarity); WHOQOL, World Health Organization Quality of Life (Subscales for domain 2—psychological health, domain 3—social relationships, domain 4—environmental); BRS, brief resilience scale; SPS-10, social provisions scale; OMSWA, opening minds survey for workplace attitudes.

**Table 3:** Valid generalized estimating equations for psychological disorder criteria screeners by modality and timepoint, using intensive modality at T1 as baseline.

		*B* (SE), [95% CI]	Wald Chi-Square	*df*	*p*
Probable PTSD—PCL5	Intercept	1.35 (0.25), [0.84, 1.86]	27.358	1	<0.001***
	Classroom	0.001 (0.32), [−0.64, 0.64]	0.000	1	0.997
	T2	−0.14 (0.26), [−0.66, 0.38]	0.277	1	0.598
	T3	0.41 (0.37), [−0.32, 1.15]	1.203	1	0.273
	T4	−0.25 (0.42), [−1.08, 0.57]	0.371	1	0.542
Depression—DASS21	Intercept	2.24 (0.35), [1.54, 2.94]	39.273	1	<0.001***
	Classroom	−0.03 (0.41), [−0.85, 0.78]	0.007	1	0.931
	T2	−0.54 (0.34), [−1.21, 0.13]	2.496	1	0.114
	T3	−0.19 (0.41), [−1.01, 0.62]	0.228	1	0.633
	T4	−0.10 (0.64), [−1.36, 1.15]	0.027	1	0.870
Anxiety—DASS21	Intercept	2.35 (0.36), [1.64, 3.06]	42.078	1	<0.001***
	Classroom	−0.36 (0.41), [−1.17, 0.45]	0.754	1	0.385
	T2	−0.46 (0.32), [−1.09, 0.16]	2.057	1	0.152
	T3	0.08 (0.41), [−0.73, 0.89]	0.038	1	0.846
	T4	−0.342 (0.54), [−1.41, 0.73]	0.390	1	0.532
Stress—DASS21	Intercept	2.56 (0.38), [1.80, 3.31]	44.033	1	<0.001***
	Classroom	−0.34 (0.40), [−1.14, 0.44]	0.736	1	0.391
	T2	−0.67 (0.37), [−1.41, 0.06]	3.239	1	0.072
	T3	−0.46 (0.42), [−1.29, 0.36]	1.207	1	0.272
	T4	−0.23 (0.60), [−1.41, 0.95]	0.147	1	0.702
Hazardous Alcohol Use	Intercept	0.83 (0.24), [0.34, 1.31]	11.264	1	<0.001***
	Classroom	1.25 (0.33), [0.59, 1.92]	13.757	1	<0.001***
	T2	−0.109 (0.28), [−0.67, 0.45]	0.143	1	0.705
	T3	0.872 (0.49), [−0.10, 1.84]	3.066	1	0.080
	T4	0.652 (0.67), [−0.68, 1.98]	0.922	1	0.337

T1, Before BOS; T2, After BOS; T3, 1 month follow-up; T4, 3 month follow-up. B, beta value; SE, standard error; CI, Wald 95% confidence interval. Chi-Square, test statistic; df, degrees of freedom. *p ≤ 0.05; **p ≤ 0.01; ***p ≤ 0.001—Statistically significant difference. Statistical significance along “Classroom” factor: difference attributable to Modality compared against Intensive. Statistical significance along “T2”, “T3” or “T4” factor: Difference attributable to Survey Timepoint compared against Intensive and Classroom at T1. PTSD, post-traumatic stress disorder; PCL-5, posttraumatic stress disorder checklist for DSM-5; DASS, depression, anxiety, and stress scale; AUDIT, alcohol use disorders identification test.

A multi-level modelling (MLM) approach was employed to assess for changes in mean self-report scores over time, and to assess whether any changes differed across delivery modalities (i.e., Intensive or Classroom, Table 4). The MLM approach provides accurate estimates when some individuals have missing timepoints (Heck et al., 2013; West et al., 2015), and also allowed for more close comparison to the previous BOS Intensive evaluation. Model fits were estimated using restricted maximum likelihood (REML) via the MIXED command in SPSS Version 28. The MLM strategy for each scale was as follows: analyses began with a two-level model consisting of a fixed effect for Time, a random intercept and slope for Time at the individual level, and the first order autocorrelation structure for the within individual covariance matrix at the repeated measures level. The random slope for Time was removed from the model due to non-convergence, which is consistent with previous BOS program evaluations using the same model criteria (Stelnicki et al., 2021). Modality was then included as a moderator variable by adding fixed effects for Modality and Time by Modality interaction. The Time by Modality interaction was not statistically significant for all scales and was subsequently removed from the fixed effects. Removing the Time by Modality interaction did not decrease model fit per a likelihood ratio test to assess the goodness of fit (−2 log-likelihood) between the reduced model without the interaction and the model that contained the interaction, as estimated with maximum likelihood. The final model reported in Table 4 contains fixed effects for Time and Modality, a random intercept at the individual level, and the autocorrelation structure at the repeated measures level estimated with REML.

**Table 4:** Two-level MLM results for overall scale totals.

	Fixed effects					ICC
	Intercept	Time			Modality	
	Average baseline level γ00 (SE), [95 %CI]	Average change (T1–T2) estimate (SE), [95 %CI], ES	Average change (T1–T3) estimate (SE), [95 %CI], ES	Average change (T1–T4) estimate (SE), [95 %CI], ES	Average change estimate (SE), [95 %CI], ES	
Probable PTSD—PCL5 Total Score	19.17 (1.50)*** [16.21, 22.13]	2.16 (1.35) [−0.50, 4.82], 0.13	−1.59 (1.48) [−4.53, 1.34], −0.09	−1.12 (1.98) [−5.04, 2.80], −0.07	2.41 (2.25) [−2.02, 6.84] 0.14	0.72
Probable PTSD—PCL5 Cluster B	4.55 (0.38)*** [3.80, 5.30]	0.29 (0.40) [−0.50, 1.09] 0.07	−0.36 (0.45) [−1.24, 0.52] −0.08	0.09 (0.60) [−1.09, 1.27] 0.02	0.58 (0.56) [−0.53, 1.69] 0.13	0.61
Probable PTSD—PCL5 Cluster C	2.28 (0.20)*** [1.90, 2.67]	0.17 (0.21) [−0.25, 0.59] 0.07	−0.07 (0.25) [−0.55, 0.42] −0.03	−0.30 (0.32) [−0.94, 0.34] −0.13	0.05 (0.29) [−0.51, 0.62] 0.02	0.57
Probable PTSD—PCL5 Cluster D	6.56 (0.57)*** [5.43, 7.69]	0.68 (0.53) [−0.37, 1.72] 0.10	−0.93 (0.65) [−2.21, 0.35] −0.14	−1.30 (0.86) [−3.00, 0.40] −0.20	0.74 (0.85) [−0.94, 2.42] 0.11	0.66
Probable PTSD—PCL5 Cluster E	5.68 (0.49)*** [4.72, 6.64]	1.15 (0.42)** [0.32, 1.98] 0.21	−0.15 (0.45) [−1.03, 0.73] −0.03	0.47 (0.59) [−0.69, 1.63] 0.08	1.21 (0.73) [−0.23, 2.65] 0.22	0.75
Depression—DASS21-Dep	8.26 (0.78)*** [6.73, 9.80]	1.22 (0.73) [−0.22, 2.67] 0.14	−0.32 (0.88) [−2.06, 1.42] −0.04	−1.45 (1.21) [−3.85, 0.95] −0.16	0.29 (1.18) [−2.03, 2.61] 0.03	0.67
Anxiety—DASS21-Anx	5.73 (0.63)*** [4.49, 6.98]	0.78 (0.66) [−0.52, 2.08] 0.11	−1.52 (0.76)* [−3.03, −0.01] −0.21	−0.47 (1.04) [−2.54, 1.60] −0.06	0.64 (0.95) [−1.24, 2.51] 0.09	0.61
Stress—DASS21Str	11.91 (0.81)*** [10.31, 13.50]	0.70 (0.72) [−0.73, 2.12] 0.08	−0.56 (0.86) [−2.25, 1.14] −0.06	−1.92 (1.17) [−4.24, 0.40] −0.21	0.80 (1.23) [−1.63, 3.23] 0.09	0.71
Emotional regulation—DERS36 Total Score	78.06 (2.11)*** [73.90, 82.21]	1.55 (1.43) [−1.27, 4.38] 0.07	−4.54 (1.76)* [−8.02, −1.06] −0.20	−5.74 (2.39)* [−10.48, −1.01] −0.25	3.68 (3.19) [−2.62, 9.97] 0.16	0.82
Emotional regulation—DERS36 Non-Acceptance	12.67 (0.51)*** [11.66, 13.68]	−0.16 (0.43) [−1.02, 0.69] −0.03	−1.01 (0.53) [−2.06, 0.05] −0.17	−1.68 (0.72)* [−3.11, −0.24] −0.29	1.32 (0.78) [−0.22, 2.86] 0.23	0.73
Emotional regulation—DERS36 Awareness	16.89 (0.48)*** [15.94, 17.84]	−0.05 (0.38) [−0.80, 0.70] −0.01	−0.92 (0.47) [−1.85, 0.02] −0.17	−1.81 (0.64)** [−3.08, −0.54] −0.33	0.21 (0.73) [−1.24, 1.66] 0.04	0.76
Emotional regulation—DERS36 Strategy	14.59 (0.54)*** [13.53, 15.64]	0.31 (0.40) [−0.48, 1.10] 0.05	−0.73 (0.53) [−1.77, 0.31] −0.12	−0.57 (0.74) [−2.04, 0.91] −0.09	0.73 (0.82) [−0.88, 2.34] 0.12	0.77
Emotional regulation—DERS36 Clarity	10.71 (0.32)*** [10.08, 11.35]	0.69 (0.28)* [0.13, 1.24] 0.19	−0.73 (0.34)* [−1.39, −0.07] −0.20	−0.77 (0.45) [−1.67, 0.12] −0.21	0.39 (0.50) [−0.58, 1.37] 0.10	0.71
Quality of life—WHOQOL Domain 2	63.88 (1.60)*** [60.73, 67.03]	−0.72 (1.07) [−2.84, 1.40] −0.04	−0.39 (1.50) [−3.35, 2.57] −0.02	1.28 (2.12) [−2.93, 5.48] 0.07	−0.80 (2.47) [−5.66, 4.06] −0.04	0.81
Quality of life—WHOQOL Domain 3	63.21 (1.78)*** [59.70, 66.72]	0.48 (1.49) [−2.46, 3.41] 0.02	−1.18 (1.82) [−4.77, 2.40] −0.06	1.39 (2.40) [−3.36, 6.15] 0.07	−2.68 (2.73) [−8.06, 2.70] −0.13	0.76
Quality of life—WHOQOL Domain 4	75.04 (1.25)*** [72.59, 77.50]	−1.48 (0.98) [−3.42, 0.46] −0.10	−2.91 (1.05)** [−5.00, −0.82] −0.21	0.67 (1.38) [−2.06, 3.40] 0.05	−1.50 (1.92) [−5.29, 2.29] −0.11	0.81
Resilience—BRS Total Score	3.71 (0.06)*** [3.59, 3.84]	−0.02 (0.06) [−0.13, 0.09] −0.03	0.03 (0.07) [−0.10, 0.16] 0.04	−0.01 (0.09) [−0.18, 0.17] −0.01	−0.18 (0.10) [−0.36, 0.01] −0.26	0.73
Support—SPS10 Total Score	32.53 (0.52)*** [31.50, 33.55]	−0.28 (0.59) [−1.45, 0.89] −0.05	1.19 (0.64) [−0.08, 2.46] 0.20	0.13 (0.82) [−1.49, 1.75] 0.02	−0.19 (0.78) [−1.73, 1.36] −0.03	0.59
Stigma—OMSWA Total Score	14.06 (0.49)*** [13.10, 15.02]	0.67 (0.56) [−0.44, 1.77] 0.13	0.93 (0.68) [−0.42, 2.28] 0.19	−0.11 (0.88) [−1.85, 1.63] −0.02	1.80 (0.71)* [0.41, 3.20] 0.36	0.55

SE, standard error; CI, 95% confidence interval; ICC, intraclass correlation; PCL-5, posttraumatic stress disorder checklist for DSM-5 (subscale cluster B—re-experiencing, cluster C—avoiding, cluster D, negative thoughts, cluster E—arousal/alertness); DASS, depression, anxiety, and stress scale; DERS, difficulties in emotion regulation scale (subscales for non-acceptance of emotional responses, lack of emotional awareness, limited access to emotional regulation strategies, lack of emotional clarity); WHOQOL, World Health Organization Quality of Life (Subscales for domain 2—psychological health, domain 3—social relationships, domain 4-environmental); BRS, brief resilience scale; SPS-10, social provisions scale; OMSWA, opening minds survey for workplace attitudes. *p < 0.05, **p < 0.01, and ***p < 0.001. The AUDIT is not reported due to insufficient variance in the measure to estimate the model.

Random effects (i.e., between person variance in baseline level time *τ*00, and within individual variance *σ*^2^) were statistically significant for all models (*p*s < 0.05) and were omitted from the final table for brevity. The random effects estimates were used to calculate intraclass correlation coefficients included for interpretation. Standardized effect sizes (ES) and intraclass correlation coefficients (ICC) for the MLM analysis were calculated manually using estimates of fixed and random effects. Standardized effect sizes were calculated for each fixed effect using a pooled variance estimate from the final model for each scale. Using pooled variance to standardize effect size estimates accounts for the within and between individual variance in repeated measures designs (Pustejovsky et al., 2014; Westfall et al., 2014). The standardized effect sizes can be interpreted like Cohen’s *d* (Pustejovsky et al., 2014), in which 0.20 represents a small effect, 0.50 represents a medium effect, and 0.80 represents a large effect (Pleil et al., 2018). ICCs were calculated for each applicable scale or subscale using the between individual (random intercept) variance estimate and the residual variance estimate from the empty random intercept model. The empty random intercept model consisted of a random intercept at the individual level, and no fixed effects, estimated with REML. In the absence of model predictors, the ICC provides an estimate of the proportion of variance attributable to individual differences (Raudenbush and Bryk, 2002; Hox and De Leeuw, 2003).

## 3 Results

Sociodemographic characteristics of participants in both modalities were largely comparable (Table 1). The only statistically significant effect of modality for positive screens indicated Classroom modality participants at pre-training had a lower prevalence of positive screens for potentially hazardous alcohol use than Intensive modality participants (Table 3). The differences in prevalence of positive screens for mental health disorders between the modalities were largely attributable to individual differences between participants (Table 3).

There were no statistically significant differences between modalities for other measures, except that OMSWA total scores were statistically significantly higher (*p* < 0.05) among Intensive participants (Table 4). Average change estimates (Table 4) indicated PCL-5 Cluster E (hyper arousal/alertness) scores increased statistically significantly (*p* < 0.05) for all participants from pre- to post-training. Anxiety and environmental quality of life subscale scores decreased (*p* < 0.05) for all participants from pre-training to 1-month follow-up. Emotional regulation total scores decreased from pre-training to 1 month follow-up (*p* < 0.05), and pre-training to 4-month follow-up (*p* < 0.05) (Table 4). Emotional regulation subscale scores decreased (*p* < 0.05) for “Non Acceptance of Emotional Responses” and “Emotional Awareness” from pre-training to 4 month follow-up. “Emotional Clarity” subscale scores evidenced a statistically significant increase from pre- to post-training, and a statistically significant decrease from pre-training to 1 month follow-up. Decreases in resilience from pre- to post-training, and from pre-training to 4 month follow-up, and increases in resilience from pre-training to 1 month follow-up were not statistically significant.

All model random effects were statistically significant (*p*s < 0.05), indicating a large proportion of the variance observed in outcome measures across time was attributable to initial differences between individuals, as well as differences within each individual. The results were supported by medium to high ICC values associated with all outcome measures.

### 3.1 Qualitative analyses results

The most heavily emphasized theme across participant responses was *insight and awareness changes*, with many participants describing thinking about themselves and their experiences in new ways (cognitive); behaving in different ways or acting upon new awareness (behavioural); and understanding their feelings and emotional responses, including being more attuned to bodily sensations (emotional). Participants in both modalities described increased awareness or insight within cognitive (Intensive: *n* = 11; Classroom: *n* = 24), emotional (Intensive: *n* = 13; Classroom: *n* = 6), and behavioural domains (Intensive: *n* = 9; Classroom: *n* = 5). Intensive participants more frequently described increased emotional and behavioural awareness, whereas Classroom participants more frequently described increased cognitive awareness. For example, one Intensive participant described increased attention to and recognition of their emotional experiences: “I have been able to recognize certain emotions as they are occurring and work through them, opposed to before when I would just become angry because I did not understand the emotion” (Participant 17). Intensive (*n* = 5) and Classroom (*n* = 2) participants also described changes in their familial relationships: “I’ve started communicating better with my wife and being able to relate and explain some of her feelings and thoughts to what I’ve learned in the program as well” (Intensive Participant 15).

The *stigma reduction* theme included participant descriptions of feeling less different from others or feeling less alone with their mental health symptoms, either via the explicit learning content (i.e., learning about brain-based or physiological responses to PPTE or other stressful events) or via group discussion of said content (i.e., relating to others’ experiences, experiencing normalization, and validation of responses to trauma or stress by sharing and hearing others’ stories). Intensive (*n* = 14) and Classroom (*n* = 4) modality participants described reductions in stigma via the group dynamic: “I have found a small cadre of others in the same boat as me, people I can relate with who had also stepped forward to say ‘I’m broken’” (Intensive Participant 4). Classroom (*n* = 7) and Intensive (*n* = 2) modality participants described the learning content contributed to reductions in feelings of isolation: “The program has been very helpful, but the one thing that really was … [that] PTSD is not a mental illness but a brain injury.” (Classroom Participant 39). This variation indicates that while both modalities were perceived as beneficial, consistent with the program content emphasized in each modality, Intensive participants more frequently cited group discussions and Classroom participants discussed the learning content.

The *delivery* theme included participant descriptions of program accessibility, quality, and delivery of the program. Classroom modality participants (*n* = 17) expressed negativity towards the delivery method, indicating a preference for Intensive delivery. There were also Classroom modality participants (*n* = 18) who reported feeling they had missed out on the benefits of group sessions and described online participation as difficult: “… very hard to express your feelings and receive feedback… The program would be far more beneficial if it was in a group setting, face to face with others” (Classroom participant 52). Intensive modality participants (*n* = 8) more often described participation challenges such as scheduling of the sessions and time required to attend compared to Classroom participants (*n* = 2).

## 4 Discussion

The current study was designed to evaluate the effectiveness of the virtual modality of the BOS program (BOS Classroom) as compared to the existing in-person delivery (BOS Intensive). The current results support the Classroom modality as generally comparable to the Intensive modality in terms of changes in self-reported mental health symptom outcomes. Across both modalities, the BOS program was associated with small, but statistically significant, improvements in self-reported anxiety and emotional regulation. The current results are consistent with a previous evaluation of BOS Intensive (Stelnicki et al., 2021), but incrementally evidence some of the small, statistically significant improvements in anxiety and emotional regulation, some of which were sustained 4 months after completing the training.

Consistent with our hypotheses, outcomes following the BOS program were largely comparable across both Classroom and Intensive modalities, with no differences in effects observed between modalities. The only modality effect was for the OMSWA measure, which differed statistically significantly between groups at initial scores, indicating changes in outcome measures did not meaningfully differ between delivery types. The proportion of participants who screened positive for mental health disorders were also largely comparable across modalities, except for a higher prevalence of potentially hazardous alcohol use positive screens among Intensive participants at pre-training. There were no statistically significant decreases in the number of participants who screened positive for mental health disorders over time after controlling for individual differences, suggesting any changes reflected dynamic individual circumstances, differences in relative engagement with the BOS material, or other unmeasured variables. The results were consistent with previous research evidencing Mindfulness-based Stress Reduction training as comparable across virtual and in-person formats among US military personnel and Veterans (Rice et al., 2018, 2019).

There were small, statistically significant improvements in anxiety symptoms and difficulties with emotional regulation across both modalities, which were sustained 4 months after the training. Open-text responses corroborate and contextualize the quantitative results, with participants from both modalities reporting new insights into their own thoughts and emotions concerning stressful situations. There were no statistically significant reductions in mental health stigma and feelings of isolation for participants in either modality, but participants who provided qualitative feedback described stigma reductions as important elements of the BOS program experience. Classroom modality participants primarily attributed reductions in self-stigma to the didactic component of BOS, whereas Intensive modality participants attributed the same reductions to group sharing. The feedback is consistent with prior research regarding the perceived potential impact of psychoeducation (Ricciardelli et al., 2020) and social validation (Kosmicki and Glickauf-Hughes, 1997; Cox et al., 2017; Yalom and Leszcz, 2020) for reducing stigmatizing beliefs. Reports of reduced stigma may be particularly important for PSP populations, as previous research has identified stigma towards mental health as a primary barrier to help-seeking behaviors in PSP (Newell et al., 2022).

Open-text responses also indicated that Intensive participants valued the group discussions, whereas Classroom modality participants described technical difficulties with online discussions sufficient to describe the discussions as detrimental to the overall training. The results contrast previous evidence suggesting preferences for virtual discussions perceived as less anxiety-provoking and more accessible (Rochlen et al., 2004; Fortier et al., 2022). Despite also facing some accessibility barriers while attending the training, participants in the BOS Intensive modality collectively expressed a preference for in-person groups. The individual variability in results and preferences, juxtaposed with the comparable results across modalities, suggests PSP may be best served by self-selecting modality training options that meet their current needs and preferences.

There was evidence of statistically significant increases in hyperarousal/alertness and decreases in environmental quality of life from pre- to post-training, possibly due to increased PPTE exposures concurrent with COVID-19 pandemic onset (Heber et al., 2020). Participants reported that the BOS program increased their mental health self-awareness, which may have facilitated increased attention to, and reporting of, the progressive pandemic impacts. In general populations, 40 to 70% of those who met screening criteria for PTSD symptoms no longer screened positive after CBT treatments or interventions (Bradley et al., 2005). In military and Veteran populations, many participants still report residual symptoms after CBT and prolonged exposure therapy (Bradley et al., 2005; Steenkamp et al., 2015; Allan et al., 2017), which may indicate these particular symptoms are resistant to treatment or are more successfully addressed over longer periods of time. Hyperarousal symptoms in particular were reported to be treatment resistant to typical CBT in military and Veteran populations (Crawford et al., 2019; Schnurr and Lunney, 2019; Miles et al., 2023). Promising alternative solutions have been explored in Veteran populations, such as mediation-based intervention (Crawford et al., 2019) to address this category of symptoms. Consistent with previous research results for BOS (Stelnicki et al., 2021) there were no statistically significant changes in self-reported resilience among participants in either modality.

Existing time-limited training interventions for PSP have generally demonstrated limited effectiveness for improving mental health outcomes (Carleton et al., 2018b, 2019a; Anderson et al., 2020). The BOS program presents a promising option for PSP mental health training as a function of small, but statistically significant, improvements in measures of anxiety, emotional regulation, and mental health stigma, which appear comparable across in-person and virtual modalities. Participants in the BOS Intensive program reported challenges with accessibility, but still reported a preference for in-person engagement. In addition, the virtual BOS training may be a viable alternative for PSP who find in-person sessions less accessible or convenient. Allowing PSP to select the modality that best aligns with their current circumstances and learning preferences could lead to increased engagement and, potentially, more effective training outcomes.

### 4.1 Limitations and future directions

The current study has several limitations that can help inform future research directions. First, the Classroom modality was introduced in response to growing accessibility needs and COVID-19 pandemic safety measures, which meant that the current data were based on a quasi-experimental design without randomized assignment to each modality. Second, there is no way to differentiate the relative impact of the COVID-19 pandemic from the impact of the BOS program using data from the current sample (Yu et al., 2020; Combden et al., 2022; Bouza et al., 2023) with PSP and healthcare workers appearing to have been disproportionately impacted by the COVID-19 pandemic (Heber et al., 2020; Marchildon et al., 2020; Hossain and Clatty, 2021; Cadell et al., 2022; Xue et al., 2022; Patel et al., 2023). The comparable effects found in the two modalities despite this time delay suggest the effects associated with BOS may be sustained even in high-stress social environments. Third, there was considerable intrapersonal variability in many outcome measures, reducing the statistical power to detect additional small effects at 1 month and 4 month follow-up assessments. Future researchers should consider using explicit random assignment or participant self-selection and including measurements of skill acquisition and use as part of evaluating the relative impact of the BOS program. Fourth, there was substantial attrition during the study such that the sample size at the 4 month follow-up was modest and the associated results may reflect important self-selection biases. Fifth, the current qualitative evidence suggests important elements of participant experiences may not be entirely captured in quantitative surveys, highlighting the importance of nuanced assessment of participant perceptions of the program in program evaluations.

## 5 Conclusion

The current study evaluated BOS program delivery and expanded previous research on the BOS program by including a longer follow-up period, comparing the Intensive (i.e., in-person) and Classroom (i.e., virtual) delivery modalities, and including qualitative analyses of participant experiences. The current results were consistent with previous research and evidenced small, but statistically significant, improvements in anxiety and emotional regulation, some of which were sustained 4 months after training. Changes in mental health symptoms were largely comparable across Intensive and Classroom modalities; however, many participants reported a preference for the Intensive program despite acknowledging accessibility benefits of the Classroom modality. Participants reported perceiving stigma reductions as part of qualitative data collection that were not reflected in quantitative self-report analyses. The comparable results across modalities suggests PSP may be best served by self-selecting modality training options that meet their current needs and preferences.

## Data availability statement

The datasets presented in this article are not readily available because the dataset will be made available only to persons with the necessary clearances as defined by the research ethics board at the University of Regina. Requests to access the datasets should be directed to RNC nick.carleton@uregina.ca.

## Ethics statement

The studies involving humans were approved by University of Regina Research Ethics Board. The studies were conducted in accordance with the local legislation and institutional requirements. The participants provided their written informed consent to participate in this study.

## Author contributions

GI: Data curation, Formal analysis, Investigation, Methodology, Supervision, Writing – original draft, Writing – review & editing. NB: Data curation, Formal analysis, Investigation, Methodology, Writing – original draft, Writing – review & editing, Validation. MR: Data curation, Formal analysis, Investigation, Methodology, Writing – original draft, Writing – review & editing. AW: Formal analysis, Investigation, Writing – original draft, Writing – review & editing. AS: Formal analysis, Writing – original draft, Writing – review & editing. JD: Formal analysis, Investigation, Writing – review & editing. JK: Formal analysis, Investigation, Writing – original draft, Writing – review & editing. KB: Formal analysis, Investigation, Writing – review & editing. SW: Investigation, Writing – original draft. TC: Investigation, Writing – original draft, Writing – review & editing. TT: Data curation, Formal analysis, Investigation, Methodology, Writing – review & editing. JP: Writing – review & editing. KM: Conceptualization, Investigation, Writing – review & editing. RNC: Conceptualization, Investigation, Supervision, Writing – review & editing.
